# Understanding the Role of Semiochemicals on the Reproductive Behaviour of Cheetahs (*Acinonyx jubatus*)—A Review

**DOI:** 10.3390/ani11113140

**Published:** 2021-11-03

**Authors:** Alexia Tommasi, Jacek A. Koziel, Annelin H. Molotsi, Giulia Esposito

**Affiliations:** 1 Department of Animal Sciences, Stellenbosch University, Stellenbosch 7600, South Africa; alexia.tommasi@gmail.com (A.T.); annelind@sun.ac.za (A.H.M.); 2 Department of Agricultural and Biosystems Engineering, Iowa State University, Ames, IA 50011, USA; koziel@iastate.edu; 3 Department of Veterinary Science, University of Parma, 43126 Parma, Italy

**Keywords:** asymmetric reproductive aging, breeding, endangered species, Felidae, marking fluid, pheromones, precopulatory behaviour, reproduction, wild cats, wildlife conservation

## Abstract

**Simple Summary:**

This review aims to provide an in-depth overview of the reproductive physiology and behaviour of cheetahs (*Acinonyx jubatus*). Specifically, it focuses on the role that pheromones (a class of semiochemicals) play by directly affecting the reproductive (e.g., precopulatory and copulatory) behaviour. Furthermore, it aims to critically analyze current research and provide new insights on study areas needing further investigation. It is clear, for instance, that further research is necessary to investigate the role of semiochemicals in the reproductive behaviour of cheetahs in order to rectify the current behavioural difficulties experienced when breeding younger females. This, in turn, would aid in improving captive breeding and the prevention of asymmetric reproductive aging.

**Abstract:**

The cheetah species *(Acinonyx jubatus*) is currently listed as vulnerable according to the International Union for Conservation of Nature (IUCN). Captive breeding has long since been used as a method of conservation of the species, with the aim to produce a healthy, strong population of cheetahs with an increased genetic variety when compared to their wild counterparts. This would then increase the likelihood of survivability once released into protected areas. Unfortunately, breeding females have been reported to be difficult due to the age of these animals. Older females are less fertile, have more difficult parturition, and are susceptible to asymmetric reproductive aging whereas younger females tend to show a significantly lower frequency of mating behaviour than that of older females, which negatively affects breeding introductions, and therefore mating. Nonetheless, the experience from breeding methods used in some breeding centres in South Africa and the Netherlands, which also rely on the role that semiochemicals play in breeding, proves that cheetahs can be bred successfully in captivity. This review aims to give the reader an in-depth overview of cheetahs’ reproductive physiology and behaviour, focusing on the role that pheromones play in this species. Furthermore, it aims to provide new insight into the use of semiochemicals to improve conservation strategies through captive breeding.

## 1. Introduction

With less than 7100 cheetahs left in the world [1] and an overall classification as “*vulnerable with a decreasing population trend*” [2], the cheetah is one of many species in need of conservation efforts [1]. During the last ice age, the cheetah species almost went extinct [3] and experienced a “*population bottleneck*” that led to reduced genetic diversity, followed by high levels of inbreeding [4]. As reported by Frankham [5], the International Union for Conservation of Nature (IUCN) considers genetic diversity as “*one of the three levels of biodiversity requiring conservation*”. Therefore, captive breeding aims to create a captive population that would act as a vital genetic reservoir in the case of another sudden, unforeseen loss of wild populations [6]. Besides the fact that reproduction is a crucial part in ensuring the conservation and survival of a species [7], the main aim of captive breeding is to produce a population of cheetahs with a higher genetic variety when compared to their wild counterparts. This has become a necessity in creating a sustainable, healthy population of cheetahs that would survive after possible reintroduction into protected wild areas [8,9]. Therefore, studies focusing on increasing natural reproduction in supervised populations are crucial to obtaining a self-sustainable cheetah population. 

Research advancement in animal behaviour related to reproduction, raising of offspring, breeding, and communication, including that of chemical communication (the predominant form of interaction between many organisms) [10], is crucial for the conservation of numerous endangered animals [11].

Cheetahs rely on their advanced olfactory systems for chemical communication [12] which contributes largely to regulating animal behaviour [13], including sexual receptivity [10]. Chemical communication occurs through volatile chemical signals and/or non-volatile chemical signals. The former is generally fixed by lipids, released into the air, and detected as an odour, whereas non-volatile chemical signals are transferred to the vomeronasal organ of an animal’s olfactory system [14,15]. The odours that are released bind to corresponding receptors on the sensory neurons to stimulate a response; thus, the shape and structure of the chemical compounds are important in the discrimination of different odours [12]. The release and reception of chemical signals (semiochemicals) from one animal to another is involved in a very intricate system from initial detection to final response [16] and contributes largely to regulating animal behaviour [13], including sexual receptivity [10].

Therefore, this review aims to provide the reader with an overview of the cheetah’s characteristics and, in particular, on its reproductive behaviour focusing on the role that semiochemicals play in this species. Furthermore, it aims to provide new insight into the use of semiochemicals to improve conservation strategies through captive breeding.

## 2. The Cheetah

Well-known for its speed, the cheetah (*Acinonyx jubatus*) is a medium-sized feline and the only member of its genus [17]. In the past, the cheetah could be found throughout Asia and Africa. Today, however, they can only be found in Iran and sub-Saharan Africa in grassland and savannah habitats, with larger populations in areas with an abundance of prey and a large stretch of land [12].

In the 1900s, the cheetah population was composed of over 100,000 individuals worldwide. Today, less than 7100 cheetahs are left [1], with a 76% loss in Africa and a reduction of the Asian population to very small numbers [6]. The Asian population is therefore listed as critically endangered [6] and the overall cheetah population is listed as vulnerable with a decreasing population trend [2]. The decreasing trend emphasizes the need for more effort in conserving the species [6]. 

### 2.1. Threats

Some of the leading causes relating to the endangerment of many wild cat species, including tigers and leopards, are the disturbance humans have on the environment [18,19,20], along with harmful human activities such as poaching and climate change [11,17]. With regards to cheetahs, the increasing human population and, therefore, the utilization of more land for activities such as farming [21] has led to the fragmentation or total loss of habitat [17] and has resulted in a total loss of 91% of historic range since the 1900s [1]. Competition, therefore, arises between farmers and cheetahs, where cheetahs are killed because they are perceived as pests and a threat to livestock [12,22].

As an intermediate predator, a cheetah faces a great deal of competition from larger predators [23]. Unfortunately, with cheetahs being built solely for speed there is the disadvantage of insufficient physical strength to protect its offspring, territories, and prey from stronger predators, predominantly lions and hyenas [24,25,26,27]. Therefore, a decline in habitat would cause an increase in the abundance of these predators in certain areas, thus causing cheetahs to avoid specially protected areas that serve as a refuge for most large predators [12].

### 2.2. Possible Reasons for Population Decline

Generally, felids have small litter sizes (1–5 with an average of 3) because females usually have no aid from males or other females in hunting, providing and caring for their young, and later teaching their offspring how to hunt [25]; in contrast, cheetahs have large litter sizes (1–8 with an average of 4) [22,27], with low birth weights (approximately 380–700 g) [27] and high growth rates. These traits, along with the swift return to oestrus after the loss of an unweaned litter of cubs, have been speculated to have evolved as a response to the high mortality rate of cheetah cubs in order to increase survival rate [23]. 

Studies evaluating cub mortality and possible causes, showed that the most common causes of death were predation [23,28], abandonment [23] starvation and injury [28]. Unfortunately, cheetah cub survival in predator-free, unprotected areas, is not much greater than in protected areas containing large predators [28] because cheetahs inhabiting unprotected areas are more prone to human conflict, including the illegal pet trade [22].

In captivity, the highest amount of cub deaths is attributed to bad mothering ability [29]. In the wild, the ability to find and obtain prey plays a primary role in the abandonment of cubs by their mothers [23]. In captivity, however, it could be speculated that a contributing factor most likely responsible for the high cub mortality could be related to the mother’s genetics. 

Several studies have proved that mothering ability is a genetically linked trait, and it is essentially related to the rate of offspring survival before weaning [30,31,32,33,34]. While it is possible to select for good mothering ability in a captive breeding program, selective breeding is difficult for species with a low genetic diversity [35].

Cheetahs population is said to have undergone a genetic bottleneck that severely reduced genetic diversity [36], followed by constant inbreeding [4]. It has been known for a long time that inbreeding reduces survival and reproduction in a species, including mothering ability, sperm production, and adult and juvenile survival [5]. 

### 2.3. Reproductive Characteristics

Female cheetahs reach sexual maturity at 20–24 months of age [1] and have a considerably short oestrous cycle if compared to those of other large felids, which lasts 20–30 days [24]; in fact, the duration of the oestrous cycle is between 7–21 days, with oestrus lasting between 2–6 days on average and inter-oestrous periods lasting for 13.9 ± 0.7 days [17,37]. 

Cheetahs are induced ovulators [9], meaning that frequent copulation contributes in maintaining high levels of oestrogen, and, in combination with the low levels of progesterone, stimulates the anterior pituitary to release luteinizing hormone (LH) and trigger ovulation [38,39,40,41,42].

Female cheetahs are also polyoestrous; therefore, oestrus and breeding occur throughout the year [9,12,24]. In the wild, however, it has been seen that females “decide” when it is the right time to come on oestrus [12] and are therefore thought of as being seasonally polyoestrous [22]. In captive breeding facilities, with no environmental pressure, reproduction and oestrous onset can occur at any time throughout the year [6]. At 6 years of age, females reach maximum reproductive capability, which continues until they are 8 years old and declines thereafter [22].

Unfortunately, female cheetahs are susceptible to asymmetric reproductive aging (ARP); therefore, the older a female is when she has her first litter, the shorter her reproductive lifespan will be. This occurs due to continuous oestrous cycling, where the fluctuating levels of oestrogen cause the reproductive organs to age at a faster rate [8]. Failure to reproduce after a long period of not reproducing is observed in a wide variety of species [43]. In female cheetahs it has been observed that although older cheetahs produced less recoverable oocytes and ovarian follicles, the quality of the gametes and function of the ovaries were unexpectedly normal. The main factor being the cause of infertility in older cheetahs was seen to be uterine integrity, with endometrial hyperplasia seen in 50% of the females considered to be in their “prime” years (e.g. best years fo reproduction; 6–8 years) and in more than 85% of the older females (9–15 years) [44]. These results are similar to that of another study with 23 species of felids that had been exposed to progestins for at least 6 months, which is believed to have stimulated the endometrial epithelial cells to start differentiating [45]. 

This rapid aging process can be prevented by breeding females when they are still young adults [8], which would also increase their chances of being able to reproduce for the rest of their reproductive lifespan [43].

### 2.4. Reproductive Behaviour

Although cheetah females display characteristic feline oestrus behaviour, there is a large amount of variability between individuals [9]. The periods in which a female cheetah will accept a male to mate with her is also very short, difficult to identify or lacking [46]. A few authors have hypothesized this as “silent” oestrus [47,48,49]. In captive breeding programs, silent oestrus is seen in younger, more inexperienced females. Therefore, even though an active male can still detect a female on oestrus by her scent, if she does not display oestrus behaviour, he will not be able to find her [50].

According to Wielebnowski and Brown [9], in cheetahs, the increased frequency of certain behaviours is correlated with increasing concentrations of faecal oestradiol and, therefore, to oestrus. In this study, they classified seven females as breeders, four as non-breeders, and three as undetermined. The breeders had all previously given birth and were older than the age of 6 years. The non-breeders were younger than 4 years and had never bred before, despite being introduced to two different males more than 15 times. The undetermined group consisted of one 2-year-old female and two 5-year-old females who had never been introduced to a male. Although this study had few limitations (females were usually housed together; they were under different management systems; the undetermined group was classified as non-breeders during data analyses), it did have interesting results. Based on their observations, in fact, the behaviours “rub”, “object sniff”, “roll”, “urine-spray” and “meow-chirp” (refer to Wielebnowski and Brown [9] for definitions) significantly correlated in a positive manner with increases in oestradiol concentrations; all females of varying ages showed typical feline oestrous behaviour; there was a significant difference in age and breeding status with regards to the frequency of oestrous behaviour displayed, where non-breeders and younger females showed significantly less characteristic oestrous behaviour. Based on their results, the researchers concluded (i) that oestrus could not be regarded as silent since all ages displayed oestrous behaviour; (ii) the frequency of oestrous behaviour displayed increased with age; and (iii) age and experience are the two main factors that influence the frequency of oestrous behaviour. 

Based on the results and conclusions of this study, the following assumption can be made: Since all non-breeders were under 4 years of age (breeders were over 6 years of age) and had all been previously introduced to males (>15 introductions) with no success, it can be assumed that even though mating behaviour is displayed by younger females, if the frequency is not enough, mating will not occur.

Whereas the above study proved that female cheetahs do show characteristic feline oestrous behaviour, it also proved the variation between individuals regarding the frequency and type of behaviour displayed by each female. This is different from other felids, where the sequence of behavioural changes is displayed in a foreseeable manner during oestrus [6]. In a captive breeding setting, the sequence displayed by each individual would need to be analysed in order to determine the oestrous state, which is difficult and time-consuming [6].

## 3. The Physiology of Olfaction

### 3.1. The Main Versus the Vomeronasal (Accessory) Olfactory System

All mammals possess a main and an accessory (vomeronasal) olfactory system besides bats, marine mammals, and humans [51,52]. These systems contain sensory neurons that are necessary to detect semiochemicals and, therefore, scent-marks [11]. Both systems are astonishingly consistent in all species of mammals, and although there are structural differences, there are also similarities in how each system functions [12,53]. The olfactory and vomeronasal receptors are very different from each other with regards to their primary structures and location [11,16]. The receptors from each system are only able to detect certain categories of compounds [51]. For example, volatile compounds are generally detected by the olfactory system via the nasal epithelium, whereas non-volatile compounds are said to be detected by the vomeronasal organ, which is located in the nasal septum [54,55]. Although the neural pathways of each system do run parallel to each other [55], the olfactory receptors transmit signals to the main olfactory bulb, and the vomeronasal receptors transmit signals to the accessory olfactory bulb [14,53]. Both neural pathways from each system, however, are involved in managing behavioural and endocrine responses [56].

In terms of detection and response to pheromones, previous misconceptions included the fact that the vomeronasal system detected only pheromones, whereas the main olfactory system could not [53]. This is not true as some pheromones utilize the vomeronasal system while others utilize the main olfactory system [16]. Furthermore, some semiochemicals can be detected by both olfactory systems [53].

Another misconception was that the main olfactory system initiated general reproductive behaviour and the vomeronasal system moderated specific behavioural signals relevant to each sex [57]. Both olfactory systems do coincide with each other [16]. In fact, although reduced, a neuroendocrine reaction in response to certain pheromones is still seen after the removal of the vomeronasal organ [55]. For example, the mating stance, which is a behavioural response to the steroidal pheromone, androstenone, was not affected after blocking the vomeronasal duct. Only in immature mammals that do not possess a functional vomeronasal organ, reproductive behaviour is affected [16].

### 3.2. The Main Olfactory System 

The arrangement of the olfactory system is similar across species with regards to how the olfactory receptors function, the physiological activity thereafter, as well as the arrangement of the olfactory central nervous system [58]. Although the main olfactory epithelium generally detects volatile compounds [54], detection of non-volatile compounds occurs when an animal expresses behaviour that consists of direct physical contact [56]. 

The two kinds of receptors in the main olfactory epithelium are known as the olfactory receptors and the trace amine-associated receptors [11]. In rats, the suckling pheromone triggers a particular segment of the main olfactory bulb [57]. This pathway is also involved in the identification of the opposite sex [16]. The fact that both pheromones and common odours can influence the neuroendocrine system [57] is most likely due to the fact that the olfactory epithelium also contains a group of receptors dedicated to detecting pheromones [59]. These receptors are otherwise known as V1R-positive and are, in fact, a type of vomeronasal receptor [56], which means that the detection of pheromones is not a specific function of the vomeronasal system [53]. 

### 3.3. The Vomeronasal (Accessory) Olfactory System

The vomeronasal system is generally believed to be the main detector of semiochemicals, especially pheromones [56], and can detect chemical compounds of both high and low volatility [51]. Detection of pheromones via the vomeronasal system plays a large role in reproduction through the influence of sexual behaviour, as well as hormone levels involved with reproduction [55]. The vomeronasal organ, or otherwise known as the organ of Jacobson [12], is where the detection of chemical compounds occurs [60]. Cats transport non-volatile semiochemicals, such as steroid conjugates and proteins, to the vomeronasal organ by using the flehmen grimace [61]. Flehmen is a behavioural response where the lower jaw opens halfway while the cat pauses and breathes steadily [62]. The flehmen response to a scent mark seems to be universal amongst all felids, including the cheetah, domestic cat, tiger, and lion [61,63,64,65]. It is used to analyse urine [51], although males tend to show this reaction at a higher rate than females [64]. 

The vomeronasal organ is found in most terrestrial mammals [16], apart from bats and some species of primates. It is a tubular structure that is found in between the oral and nasal cavities [51], consisting of two sacs filled with fluid that forms an opening at only one end [12]. The vomeronasal receptors are the V1r receptors [53] and V2r receptors [66].

### 3.4. The Link to Olfaction and Hormone Release

The connection between the gonadotropin-releasing hormone (GnRH) and olfactory systems, as well as between mediation of sexual behavioural/hormonal responses is very intricate [16]. The GnRH neurons are ultimately the main drivers of reproductive status. They have been identified to possess bidirectional connections with neurons involved in odour and pheromone processing, such as in the olfactory cortex, medial amygdala, and posterocortical amygdalar nucleus, thus proving that signals are received from both the vomeronasal and olfactory system [55]. This connection of neurons is generally referred to as the “*hypothamalmic-pituitary-gonadal axis*” [16]. Noradrenaline and serotonin levels also act as an indication of the psychological state of the animal and thus affect the reproductive endocrinology and behaviour of the animal [57]. GnRH neurons, therefore, influence the endocrinology of the animal in correlation with the psychological state of the animal [55]. Since the GnRH neurons are involved in bidirectional connections with neurons from the olfactory systems [55], semiochemicals and scent-marking would, therefore, play a role in reproduction and the endocrinology of the animal as well.

## 4. How Is Olfaction Linked to Reproduction?

### 4.1. The Effect of Pheromones on Behaviour

Although it is true that non-chemical signals, such as hearing and visualization, the physiological state, as well as the experience the animal has had in the past, are a necessity for eliciting behavioural responses [54], it has already been stated many times that semiochemicals directly affects an animal’s behaviour [12]. This is especially behaviour related to locomotion, interactions with other animals and reproduction [11,67]. The observed effects that pheromones have on an animal’s behaviour are correlated with the animal’s endocrine status [14]. The stereotypical behaviours caused by pheromones may be an unconscious response to this particular group of odours [16].

In predator-prey relationships, prey animals will exhibit defensive behaviour and try to escape once detecting the odour of a predator [68]. The semiochemicals in the urine of a dominant animal act to suppress the physiology, as well as the reproductive behaviour (such as a decrease in the rate of marking), of the subordinate animal [14].

Pheromones involved with reproductive behaviour are used as a way of attracting the opposite sex [11] and acts as an aphrodisiac in causing the appropriate precopulatory and copulatory responses [14]. The odour of a boar increases the receptivity of a female to sexual interactions, as well as promotes lordosis in 50–60% of females in oestrus [10]. Aggressive behaviour is another known response to reproductive pheromones, although it is not always just the reproductive behaviour of an animal that is affected, but the reproductive physiology as well. Pheromones have been known to accelerate puberty [12] and stimulate the onset of oestrus [69]. 

### 4.2. Captive Breeding 

Captive breeding of cheetahs occurred in response to the rapid decrease in cheetah population numbers over the past years; therefore, it initially aimed at increasing numbers, with the management of the last free-roaming cheetahs regarded as important for the species’ overall survival [37]. Unfortunately, cheetahs were found to be difficult to breed in captivity, even when there was no difference in nutrition, health, genetics, the function of the hypophysis, seminal quality in males, and anatomy of the reproductive tract in females [6,9,12,17,24,70,71]. 

Various behavioural methods were attempted by zoological facilities to rectify this and increase breeding success for cheetahs. Whereas some of these attempts have resulted in success, not one method was proven to work at all the various facilities. The key to success was said to be based on the knowledge of the reproductive behaviour of free-roaming cheetahs [21], as well as th cheetah’s natural social conditions, such as adhering to their solitary nature [9] and keeping breeding females separate [17]. With these management methods in place, females are observed for any behavioural signs that are indicative of oestrus [70], although this has proven to be very difficult in determining when females are actually on oestrus [6] and negatively affects the occurrence of breeding introductions [17]. 

Allowing each individual animal to choose their own mate is a significant part of successful breeding [72]. By using the correct housing and breeding management methods, the Ann van Dyk Cheetah Centre in South Africa (Brits, South Africa), previously known as de Wildt, has been very successful in breeding with cheetahs. It was found that the presence of male cheetahs around the females is the best method to detect when a female is on oestrus by observing her for any changes in behaviour towards the males [22]. Males are released into a walkway known as “lover’s lane” located between the female enclosures in order to smell which females are on oestrus [50]. The presence of the males has been noted to trigger the oestrous cycle in females, with interactions between the sexes resulting in a display of oestrous behaviour after the female walks towards the males in the walkway [22]. 

Another successful breeding method used at Wassenaar Wildlife Breeding Centre in the Netherlands (Wassenaar, The Netherlands) includes the utilization of one enclosure by the male and the female in turns. They are not allowed any direct visual or physical contact at first and are each observed according to their reaction to each other’s scent. Only after a lot of interest has been shown by both to the other’s scent, and the male starts stutter-calling or “yipping”, they are allowed visual exposure to one another. Females are observed once again for any aggression towards the male before physical introductions are allowed. If the female does not roll for the male or display oestrous behaviour then they are once again separated [73].

From the above methods, it can be speculated that semiochemicals play an important role in the initial process of breeding at Wassenaar, whereas they only seem to come into play during the last stage of the breeding process at the Ann van Dyk centre. Nevertheless, the breeding results achieved from both facilities prove that cheetahs can be bred successfully in captivity without the need for assisted reproductive techniques, as well as emphasizes the role that semiochemicals play in cheetah courtship. 

## 5. Semiochemicals

Semiochemicals are defined as chemical substances released by a life form that instigates a physiological or behavioural reaction from another living organism [74,75]. The earliest and most studies on semiochemicals have been on insects [14]. Semiochemicals have since been classified according to the relationship between the individual that released the chemical signal and the individual that received the signal [14], thus resulting in changes to the behaviour and/or physiology of the receiver. Semiochemicals that act as chemical signals between individuals of the same species (intraspecific) are classified as pheromones, whereas chemical signals acting between members of two different species (interspecific) are classified as allelochemicals [12]. 

Pheromones can be both odorous, thus originating from volatile chemical compounds, or non-odorous, which are from non-volatile chemical compounds [11]. Pheromones are divided into two categories based on the response to the chemical signal, namely releasers and primers. Releasers are pheromones that result in an immediate response in the behaviour of the receiver [11] and are otherwise known as signalling pheromones involved in communication [11,12]. Primers act along with the nervous system of an animal and generate a physiological response after a longer period of time [11]; therefore, primers affect mainly the endocrine system [14]. 

### 5.1. Complex vs. Single Compounds

There has been very little evidence to support the theory that a chemical signal is reliant on a number of chemical compounds [52] and it has been shown that a single chemical compound is as efficient as a mixture of compounds in evoking a behavioural response [76]. The fact that multiple chemical compounds, with each coding for the same or similar chemical signal/behavioural response, are released in scent markings is because the interaction of the chemical compounds increases detection of the signal, memorization, and learning of different chemical signals released by different species, such as those released by predators, as well as discrimination between different chemical signals [77].

### 5.2. Semiochemicals in Communication

Semiochemicals that are involved in communication act between animals/organisms that have more advanced olfactory systems, with which they rely on more than they do with hearing or vision [11,12]. Scent-marking releases a large amount of volatile and non-volatile chemical compounds that are usually present at low concentrations. The different concentrations of chemical compounds could be important with reference to the message that is sent [78]; and could release specific characteristic odours, although exactly what compounds are inhaled and how they are analysed by the animal has not yet been fully discovered [11]. 

Territorial marking, identification of neighbours, detection of larger predators and food/prey, signalling of alarm and attraction of members of the opposite sex are all functions of scent-marking [12,14,19,54]. Scent marks, in fact, also provide information on the animal’s sex, age and the reproductive state of females [14,19], which is also an indication of whether or not she is sexually receptive [79]. Genetic relatedness and compatibility of future mates can also be assessed from scent marks [54]. 

### 5.3. Identification of Individuals through Semiochemicals

Determining whether individuals have their own specific body odour, or ‘biochemical fingerprint’ or not, has become a topic of increasing interest since the identification of individuals through semiochemicals is of great significance [54]. Soso, [11] stated that semiochemicals in exocrine secretions (scent marks), which can be complex or simple mixtures, code for individuality based on the amount and the presence of particular chemical compounds. This has also been confirmed in humans where volatile compounds, identified from the sweat, urine and saliva, were shown to be both individual and gender-specific [80]. It has also been demonstrated that dogs could discriminate between the body odours of several different humans. This study showed that the dogs were able to differentiate the body odours of not only individuals of the same family, but between a set of twins as well [81]. In previous studies done with lions, over 50 volatile chemical compounds were identified in the marking fluid, with each individual having a different chemical composition [82]. Seven chemical compounds were found in the marking fluid of almost all the lions, and therefore, it is possible that some, or all of these compounds, are involved in species identification, either individually or in a combination [78].

### 5.4. Semiochemicals in Cheetahs

Although many studies have proved the importance of semiochemicals in domestic and wild felids [11,14,54,62,63,64,78,83], very little is known about their role in cheetahs. 

Unfortunately, cheetah urine itself has not acquired much interest from scientists, probably due to the very little amount of odour it emits [26]. As reported by Visser [12], who analysed the urine of three males and two females, and Burger et al. [26], who analysed the urine of six males and one female, there was a much wider variety of volatile constituents identified in female urine than male urine. The constituents that were common in the males from both studies included that of 2-butanone, phenol, hexanal, octanal, nonanal, benzaldehyde, 2-pentanone, 2-hexanone, 2-heptanone, 3-pentanone, 4-heptanone, 3-hexanone, acetophenone, cyclohexanone, 3-methylcyclopentanone, ethyl propyl ether, butyl ethyl ether, butyl propyl ether, dimethyl disulfide, dimethyl sulfone, sulfur S_6_, sulfur S_8_ and urea. Of these compounds, only a few are known to be involved in certain behaviours in various species such as 2-butanone, phenol, octanal, nonanal, benzaldehyde, 2-pentanone, 4-heptanone, acetophenone, cyclohexanone, 3-methylcyclopentanone and dimethyl disulfide [10,11,51,67]. Many of the abovementioned compounds have been identified in the marking fluid/urine of various other feline species as well. These compounds, together with the animals in which they have been found and the behaviour they elicit are listed in Table 1.

### 5.5. Current Uses of Synthetic Scent to Influence Behaviour and Its Potential Use in Cheetahs

Providing enrichment for captive animals aims mainly to improve the welfare of the animals [89] by decreasing stress and improving natural and, therefore, reproductive behaviour as well [90]. Pheromones are used quite frequently in captive facilities as a form of olfactory enrichment by introducing new scents or objects that have been scented to an animal’s enclosure [91]. Felids display cheek rubbing behaviour as a result of the use of novel scents (e.g., perfume for behavioural enrichment in zoological parks) [92]; lions respond to olfactory enrichment by behaving more socially and displaying an increase in the activity and frequency of various behaviours [76]. Besides enrichment, semiochemicals have also been specifically used for reproductive purposes in some species. For instance, synthetic boar pheromone is used to identify when a sow is on oestrus. These pheromones are already produced and sold on a commercial level in aerosol cans [51]. A synthetic scent known as Feliway^®^ has proven useful in lowering the level of corticosteroid metabolites in the faeces of tigers after artificial insemination operation procedures [67]. 

With regards to cheetahs, since the presence/odour of a male cheetah has been noted to trigger the oestrous cycle in the females [22,73] and the scent is known to play an important role in reproduction with cheetahs as well as other felids [21,39,62,63,70,73,88,93], there is an opportunity to further investigate the role of semiochemicals in cheetahs and their possible use to improve oestrus behaviours for successful mating. For instance, it could be possible to use a synthetic or natural scent in cheetahs as well in order to increase the frequency of oestrus behaviours displayed in younger females to ensure successful mating when the females are introduced to the males. 

## 6. Conclusions 

The overall cheetah population is currently listed as vulnerable, with a decreasing population trend [2]. The management of the last free-roaming cheetahs is therefore regarded as important for the species’ overall survival [37]. Captive breeding of cheetahs occurred in response to the rapid decrease in numbers in the past and therefore is aimed at increasing cheetah population numbers with a higher genetic variety when compared to their wild counterparts. This would lead to the creation of a sustainable, healthy population of cheetahs that would be able to survive after possible reintroduction [8,9,37]. Despite the fact that reproduction in wild cheetahs is not an issue, cheetahs were found to be difficult to breed in captivity [6,7,9,12,17,23,24,71,94]. 

The key to success is based on the knowledge of the reproductive behaviour of free-roaming cheetahs [21] and, therefore, managing individuals according to their natural social conditions [71]. This would include keeping males and females in separate enclosures and only introducing pairs during mating [9] and keeping breeding females separate since even small amounts of social aggression acts to suppress the oestrous cycle [17]. Using male cheetahs to detect when a female is on oestrus [22] and allowing each individual animal to choose their own mate also played a significant part in ensuring successful breeding [72].

Since female cheetahs are susceptible to the effects of asymmetric reproductive aging (ARP), it would be necessary for them to breed when they are still young adults in order to prevent this aging process [8], such as after reaching sexual maturity at 20–24 months of age [1]. Unfortunately, females younger than the age of four years display a significantly lower frequency of characteristic oestrous behaviour than females older than the age of six years [9], this means that even though a male is still able to detect a female on oestrus by her scent, if she does not display oestrus behaviour, he will not be able to find her [50] and the chance of mating is severely reduced.

Intraspecific semiochemicals [12] that are involved with reproductive behaviour are used as a way of attracting the opposite sex [11] and acts as an “aphrodisiac” in causing the appropriate precopulatory and copulatory responses [14]. These semiochemicals are synthesized by an animal [54] and released as exocrine secretions to create a ‘scent-mark’ [11], such as urine and marking fluid [67].

Since the presence/odour of a male cheetah has been noted to trigger the oestrous cycle in the females [22,73] and the scent is known to play an important role in reproduction with cheetahs as well as other felids [7,21,39,62,63,73,88,93], if one were to analyse the chemical compounds in the marking fluid of male cheetahs (with a specific focus on high impact odorants), it could lead to the identification of a single compound (or a limited number of compounds in a mixture) that would be able to increase the frequency of reproductive behaviour displayed by the females in the hopes that intercourse and pregnancy would occur at a higher rate.

## Figures and Tables

**Table 1:** Compounds identified in scent marks known to be involved in certain behaviours in several species.

Chemical Compound	Cat	Lion	Cheetah	Leopard	Tiger	Other Animals	Behaviour
Octanal		MF/U ^c,d,e^	U ^f,g^		MF ^k^	African wild dogs ^c^	Immobility ^c,e^
1-Octanol		MF ^c,e^			MF ^k,l^		Foraging, Alarm recruitment, Sensory perception ^c,e^
2,5-Dimethylpyrazine		MF ^c,e^			MF ^l^	African wild dogs, Wolf, House mouse, Tree shrew ^c,o,p^	Fear, Freezing, Aggression, Lengthens oestrus cycle, Delays puberty in both sexes, Overmarking ^c,e,o,p^
2-Butanone		MF/U ^c,d,e^	U ^f,g^		MF ^l^		Sexuality ^c,e^
2-Nonanone		MF ^c,e^	U ^f,g^		MF ^k,l^		Sex attraction ^c,e^
2-Pentanone		MF/U ^c,d,e^	U ^f,g^				Reproduction, Concentration ^c,e^
3-Methylbutanal		MF/U ^c,d,e^			MF ^l^		Attraction ^c,e^
3-Methylcyclopentanone		MF ^c^	U ^f,g^			Badgers ^c^	Seasonal reproduction ^c^
4-Heptanone		U ^d^	U ^f,g^		MF ^l^	Red fox ^p^	Overmarking ^p^
4-Methylphenol		MF ^c,e^			MF ^l^	Wild Iberian wolves, Horse ^c,q,p^	Sexuality, Estrus, Oestrus, Diestrus, Sexual attraction ^c,e,q,p^
Acetaldehyde		MF ^c,e^		MF ^i,j^	MF ^m^		Locomotion, Taste aversion, Anxiety ^c,e^
Acetic acid		MF ^c,e^	MF ^h^	MF ^i^	MF ^m,j,k^	African wild dogs ^c^	Detector of estrus, copulation, Oestrus, Attraction, Flight ^c,e^
Acetone		MF/U ^c,d,e^		MF ^i,j^	MF ^m,l^		Locomotion, Sexuality, Irritation ^c,e^
Acetophenone		MF ^c,e^	U ^f,g^		MF ^k^	African wild dogs, Fox ^c,p^	Anti-attraction, Attraction, Responsiveness, Stimulates overmarking ^c,e, p^
Benzaldehyde		MF/U ^c,d,e^	U ^f,g^		MF ^k,l^	African wild dogs ^c^	Oviposition, Defensive, Aggression, Alarm recruitment, Affects sexual behaviour ^c,e, r^
Butyrolactone		MF ^c,e^					Appetite, Vomiting, and Tremor Suppression, Estrus ^c,e^
Cyclohexanone		MF ^c,e^	U ^f,g^			Asian elephant ^p^	Attraction, Locomotion, Stimulation, Inhibition ^c,e,p^
Dimethyl disulfide		MF/U ^c,d,e^	U ^f,g^		MF ^l^		Oviposition inhibition, Attraction, Sniffing ^c,e^
Dodecan-4-olide					MF ^k^		Overmarking behaviour ^k^
Dodecanal		MF ^c,e^				African wild dogs ^c^	Physiological responses ^c,e^
Heptanal		MF/U ^c,d,e^			MF ^k^		Aggregation, Inhibited behavior, Excitation ^c,e^
Indole	U ^a^	MF ^c,e^			MF ^l^	African wild dogs, wild Iberian wolvs ^c^	Sexuality, Age differentiation ^c,e,a^
Linalool		MF ^c,e^					Alarm recruitment, Attraction ^c,e^
Nonanal		MF/U ^c,d,e^	U ^f,g^		MF ^k,l^	African wild dogs ^c^	Sexual attraction ^c,e^
Phenol		MF ^c,e^	U ^f,g^		MF ^k,l^	African wild dogs, wild Iberian wolves ^c^	Estrus, Oestrus, Sexuality ^c,e^
Phenylacetic acid					MF ^k^	Male Mongolian gerbil ^p^	Overmarking ^p^
Propanoic acid					MF ^k^	Rat ^p^	Signals estrus, Induces erections ^p^
Squalene					MF ^k^	Rat ^p^	Attraction ^p^
Trimethylamine		MF/U ^c,d,e^		MF ^i^	MF ^n,l^	Mouse ^p^	Species-specific attractant ^p^
Valeric acid	VS ^b^		MF ^h^	MF ^i^	MF ^m,j,l^		Attractiveness, Greatly affects behaviour (males and females), Estrus, Oestrus onset ^b^

U: Urine; VS: Vaginal secretions; MF: Marking fluid. ^a^ Starkenmann et al. [84]; ^b^ Bland [63]; ^c^ Soso and Koziel [67]; ^d^ Andersen and Vulpius [78]; ^e^ Soso [11]; ^f^ Burger et al. [26]; ^g^ Visser [12]; ^h^ Poddar-Sarkar and Brahmachary [85]; ^I^ Poddar-Sarkar and Brahmachary [86]; ^j^ Poddar-Sarkar and Brahmachary [75]; ^k^ Burger et al. [79]; ^l^ Soso and Koziel [19]; ^m^ Poddar-Sarkar [14]; ^n^ Banks et al. [87]; ^o^ Coombes et al [88]; ^p^ Apps et al. [51]; ^q^ Apps [52]; ^r^ Carrel [10].

## Data Availability

Not applicable.
